# Orexin, cardio-respiratory function, and hypertension

**DOI:** 10.3389/fnins.2014.00022

**Published:** 2014-02-12

**Authors:** Aihua Li, Eugene Nattie

**Affiliations:** Department of Physiology and Neurobiology, Geisel School of Medicine at DartmouthLebanon, NH, USA

**Keywords:** orexin and orexin receptors, cardiorespiratory function, hypertension, CO2 chemoreflex, blood pressure regulation

## Abstract

In this review we focus on the role of orexin in cardio-respiratory functions and its potential link to hypertension. (1) *Orexin, cardiovascular function, and hypertension*. In normal rats, central administration of orexin can induce significant increases in arterial blood pressure (ABP) and sympathetic nerve activity (SNA), which can be blocked by orexin receptor antagonists. In spontaneously hypertensive rats (SHRs), antagonizing orexin receptors can significantly lower blood pressure under anesthetized or conscious conditions. (2) *Orexin, respiratory function, and central chemoreception*. The prepro-orexin knockout mouse has a significantly attenuated ventilatory CO_2_ chemoreflex, and in normal rats, central application of orexin stimulates breathing while blocking orexin receptors decreases the ventilatory CO_2_ chemoreflex. Interestingly, SHRs have a significantly increased ventilatory CO_2_ chemoreflex relative to normotensive WKY rats and blocking both orexin receptors can normalize this exaggerated response. (3) *Orexin, central chemoreception, and hypertension*. SHRs have higher ABP and SNA along with an enhanced ventilatory CO_2_ chemoreflex. Treating SHRs by blocking both orexin receptors with oral administration of an antagonist, almorexant (Almxt), can normalize the CO_2_ chemoreflex and significantly lower ABP and SNA. We interpret these results to suggest that the orexin system participates in the pathogenesis and maintenance of high blood pressure in SHRs, and the central chemoreflex may be a causal link to the increased SNA and ABP in SHRs. Modulation of the orexin system could be a potential target in treating some forms of hypertension.

## Introduction

Orexins, also known as hypocretins (Hcrt), are two excitatory neuropeptides, orexin A and orexin B (OX-A and OX-B) or hypocretin 1 and hypocretin 2 (Hcrt1 and Hcrt 2), produced by neurons primarily located in the lateral hypothalamic area (LHA) (De Lecea et al., 1998; Sakurai et al., 1998). Both OX-A and OX-B are derived from the same neuropeptide precursor, prepro-orexin (pp-OX or Hcrt gene). The actions of OX-A and OX-B are mediated by two G-protein coupled receptors, orexin receptor-1 (OX_1_R/HcrtR1) and orexin receptor-2 (OX_2_R/HcrtR2). Mounting evidence suggests that orexin is not only important to sleep-wake cycle and feeding regulation but also to respiratory and cardiovascular regulation. Furthermore, orexin may play role in some types of hypertension.

## Orexin and orexin receptors

The anatomical and physiological properties of the orexin system have been reviewed extensively by many recent publications (Sakurai, 2007; Tsujino and Sakurai, 2009; De Lecea, 2012). In this review we focus only on orexin functions of most relevance to cardio-respiratory regulation.

### Orexin neurons

In the central nervous system (CNS), the cell bodies of orexin neurons are strictly located in the perifornical area and lateral and dorsal hypothalamic areas (Peyron et al., 1998; Date et al., 1999; Nambu et al., 1999). Orexin neurons receive afferents from GABA, serotonin and catecholamine neurons, and interact in the LHA with neurons that produce melanin concentrating hormone (MCH) and with neurons that express the leptin receptor (LepR) (Bayer et al., 2005; Leinninger and Myers, 2008; Schone et al., 2011, 2012; Burdakov et al., 2013; Karnani et al., 2013). Serotonin (5-HT) hyperpolarizes orexin neurons through 5-HT_1_A receptor mediated G-protein-coupled inward rectifier potassium channels (GIRK) in hypothalamic slices prepared from orexin neuron/enhanced green fluorescent protein (EGFP) transgenic mice (Muraki et al., 2004). The majority of orexin-immunoreactive (OX-ir) neurons in LHA are surrounded by dense tyrosine hydroxylase-immunoreactive (TH-ir) axons (Yamanaka et al., 2006), but results of studies on the role of catecholamines in orexin neurons are inconsistent. Bayer et al. showed that noradrenaline depolarized and excited orexin neurons (Bayer et al., 2005), while Yamanaka et al. reported that catecholamines directly and indirectly inhibited orexin neurons via α2-adrenoceptor mediated activation of GIRK channels (Yamanaka et al., 2006).

**Figure 1:**
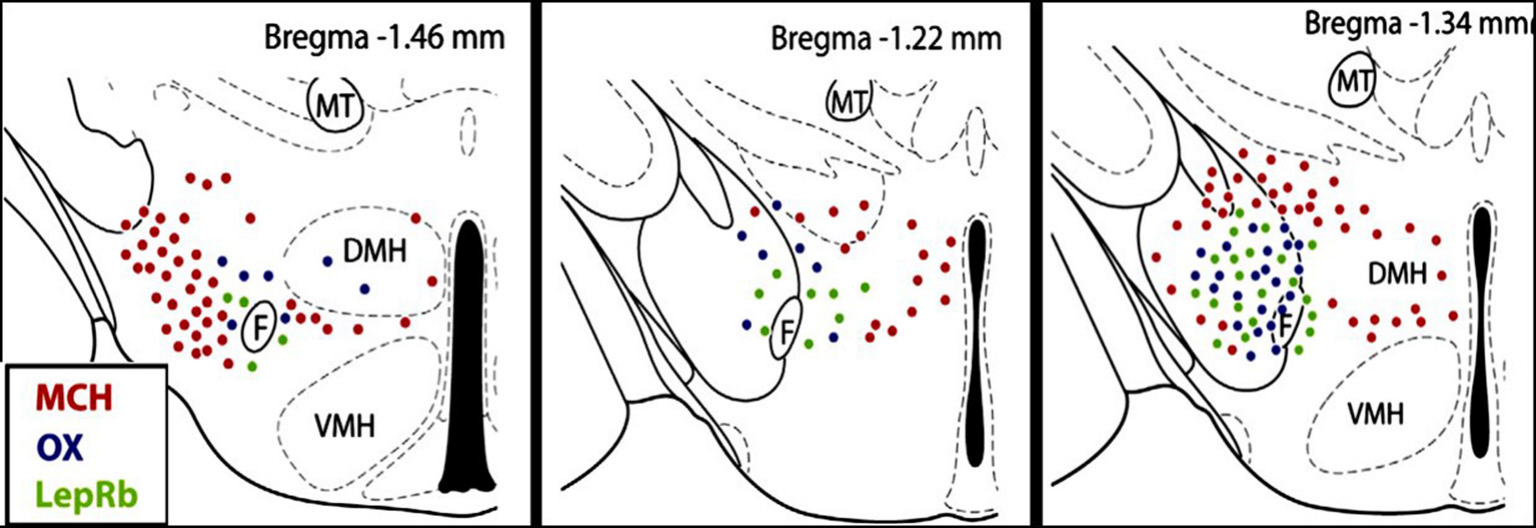
**Distribution of orexin (OX), melanin concentrating hormone (MCH) and leptin receptor (LepRb) -expressing neurons in the lateral hypothalamus (LHA)**. OX, MCH and LepRb-expressing neurons are intermingled in the LHA, but there is no co-expression. Red dots, MCH neurons; blue dots, OX neurons; green dots, LepRb neurons. F, Fornix; VMH, ventromedial hypothalamus; DMH, dorsomedial hypothalamus; MT, mammillothalamic tract (figure adapted with permission from Leinninger, 2011).

In the LHA, three major types of neurons OX, MCH, and LepRb expressing neurons are tightly intermingled and have a complex interactive relationship. An emerging hypothesis is that OX-MCH-LepR micro-circuits may act to regulate both autonomic function and energy balance (Leinninger and Myers, 2008; Hall et al., 2010; Leinninger, 2011; Burdakov et al., 2013). OX neurons are most active during wakefulness (Hassani et al., 2009), and activation of orexin receptors promotes wakefulness (De Lecea, 2010, 2012; Sakurai et al., 2010), feeding, and energy metabolism (Tsujino and Sakurai, 2009; Teske et al., 2010; Girault et al., 2012; Nixon et al., 2012), excites breathing, and stimulates sympathetic nerve activity (SNA) leading to an increase in blood pressure (Matsumura et al., 2001; Shirasaka et al., 2002; Zhang et al., 2005; Huang et al., 2010; Shahid et al., 2011, 2012; Nattie and Li, 2012). In contrast, MCH neurons are most active during sleep (Hassani et al., 2009), and MCH promotes sleep or physical inactivity (Nahon, 2006; Peyron et al., 2009; Konadhode et al., 2013; Monti et al., 2013), and regulates the autonomic nervous system. Central administration of MCH via chronic intracerebroventricular infusion (Messina and Overton, 2007) or acute injection into the nucleus of the solitary tract (NTS) (Brown et al., 2007) induces bradycardia and decreases blood pressure. Using a fluorescent-tag to identify LepRb positive neurons, Leinninger and Myers (2008); Leinninger (2011) demonstrated that in the LHA LepRb neurons do not co-express OX or MCH, but rather are co-distributed among the OX neurons (Figure 1). Both LepRb and OX neurons are surrounded by the MCH-containing neurons in the LHA (Leinninger and Myers, 2008; Leinninger, 2011). In addition, LHA LepRb neurons have direct synapses with OX neurons suggesting an important modulatory relationship between LepRb and OX neurons in LHA (Louis et al., 2010).

### Orexin projections and orexin receptors

In contract to the very localized property of the OX neurons, OX axons are widely distributed throughout the CNS, with the exception of the cerebellum (Peyron et al., 1998; Date et al., 1999; Nambu et al., 1999). Based on *in situ* hybridization both OX_1_R and OX_2_R mRNAs are distributed extensively in the same regions that contain dense OX innervation (Trivedi et al., 1998; Marcus et al., 2001), e.g., the forebrain, the hypothalamus, the brainstem and the spinal cord. Both OX receptors and efferent projections are found in many sites involved in cardiovascular, respiratory and thermo regulation, e.g., the paraventricular nucleus (PVN), NTS, retrotrapezoid nucleus (RTN), locus coeruleus (LC), Kölliker-Fuse nucleus, rostral ventrolateral medulla (RVLM), medullary raphe, lateral paragigantocellular nucleus, midbrain periaqueductal gray, A5 noradrenergic cell group, parabrachial region, area postrema, intermediolateral cell column of the spinal cord and sympathetic pre-ganglionic neurons (Peyron et al., 1998; Trivedi et al., 1998; Date et al., 1999; Nambu et al., 1999; Marcus et al., 2001). In the brainstem, networks of OX-ir fibers and terminals are expressed on the neurons of all major catecholamine cell groups [adrenaline (Adr): C1, C2, and C3 and noradrenaline (NA): locus coeruleus, A1, A2, A4, A5 and A7] (Puskas et al., 2010). Intracerebroventricular (icv) injection of orexin induces *c-fos* expression in the locus ceruleus, arcuate nucleus, central gray, raphe nuclei, NTS, supraoptic nucleus (SON), and PVN in Wistar rats (Date et al., 1999). The widespread nature and specific connections of the orexin system suggests that orexin may be involved not only in the regulation of the sleep-wake cycle and appetite but also autonomic functions, particularly cardiorespiratory functions. Orexin may act to link the regulation of cardiorespiratory functions to wakefulness and sleep.

### Summary of orexin neurons and receptors

Both orexin projections and orexin receptors are enriched in the neuronal sites that are importantly involved in cardio-respiratory regulation, and they are well positioned to participate in the regulation of cardio-respiratory functions.

## The role of orexin in cardiovascular function

### Orexin, blood pressure, and sympathetic nervous system

**Figure 2:**
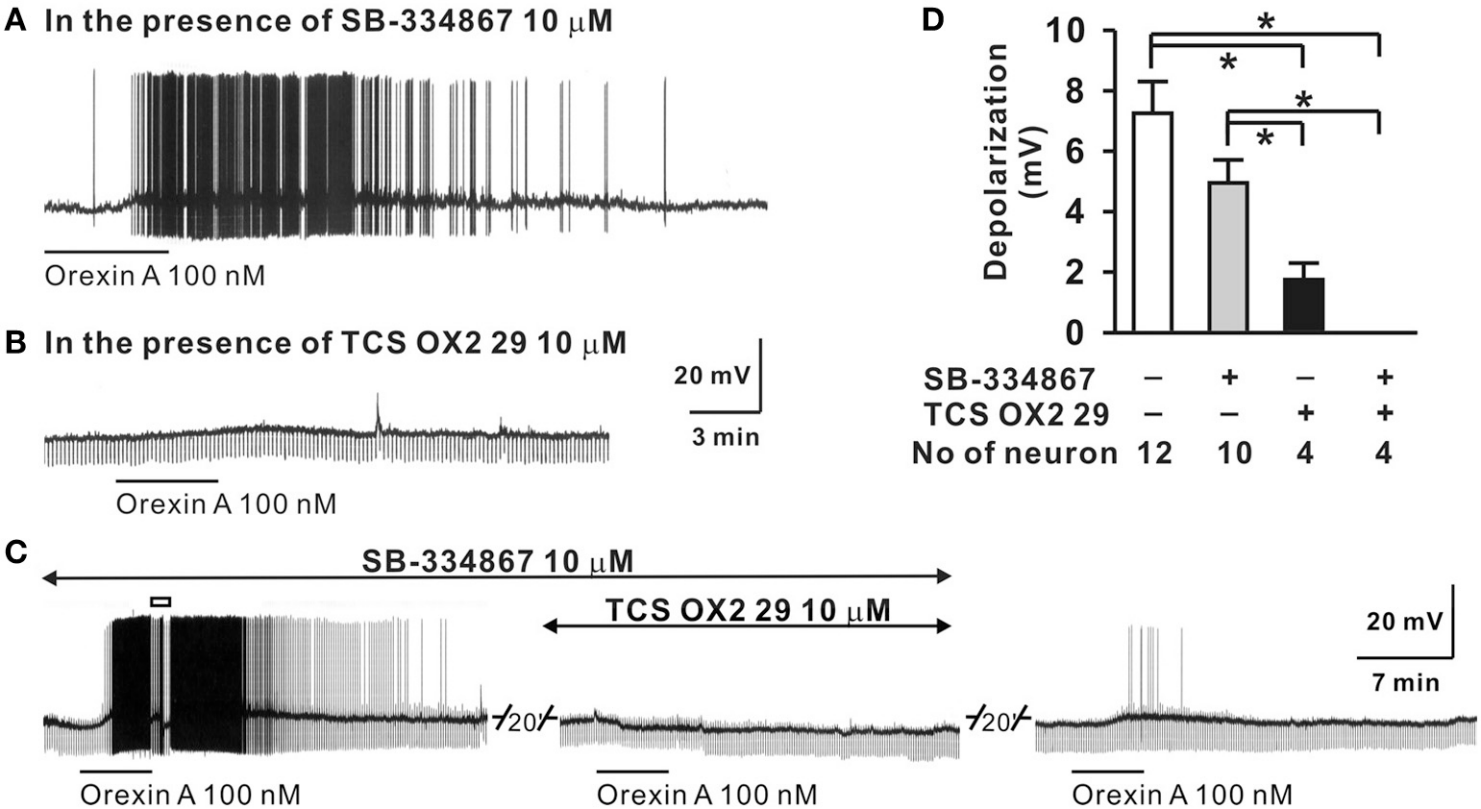
**Orexin A excites RVLM neurons *in vitro***. The OX-A induced depolarization of RVLM neurons **(D)** was: (1) not significantly affected by OX_1_R antagonist SB-334867 **(A,D)**, (2) significantly reduced by an OX_2_R antagonist, TCS OX2 29 **(B,D)**, and (3) abolished by simultaneous application of OX1R and OX2R antagonists **(C,D)**. (Figure used with permission from Huang et al., 2010). Values are the mean ± S.E.M. with the numbers of neurons indicated below each bar. ^*^Significant difference *p* < 0.05 (One-Way ANOVA followed by the Student-Newman-Keuls test).

The sympathetic nervous system (SNS) plays a crucial role in the regulation of circulation and blood pressure (Guyenet, 2006; Fisher and Paton, 2011; Zubcevic et al., 2011), and many neuronal groups in the lateral hypothaluamus and brainstem are critically involved in such regulation. It is known, that *in vivo*, electrical or chemical stimulation of the perifornical nucleus of the hypothalamus increases blood pressure and heart rate (HR) and activates neurons of the lateral paragigantocellular area (Sun and Guyenet, 1986; Allen and Cechetto, 1992). Soon after orexin and orexin receptors were discovered in 1998, many studies started to examine if orexin in the LHA participation in the regulation of cardiovascular and sympathetic functions (Samson et al., 1999; Shirasaka et al., 1999; Matsumura et al., 2001). *In vitro*, orexin in a dose-dependent manner, depolarizes neurons that are involved in the regulation of blood pressure and sympathetic nerve activity (SNA), e.g., neurons in the hypothalamic PVN and the RVLM (Shirasaka et al., 2001; Follwell and Ferguson, 2002; Huang et al., 2010), as well as spinal cord sympathetic preganglionic neurons (Antunes et al., 2001). In the RVLM both OX-A and OX-B depolarize neurons in a dose dependent manner, and at 100 nM, orexin excited ~42% of neurons in the area. Application of an OX_2_R antagonist (TCS-OX2-29) significantly reduced the number of neurons activated by OX-A, while co-application of OX_1_R and OX_2_R antagonists completely eliminated orexin A-induced depolarization (Figure 2) (Huang et al., 2010). Furthermore, about 88% of adrenergic, 43% of noradrenergic, and 36–41% of rhythmically firing RVLM neurons can be excited by orexin in the RVLM (Huang et al., 2010).

**Figure 3:**
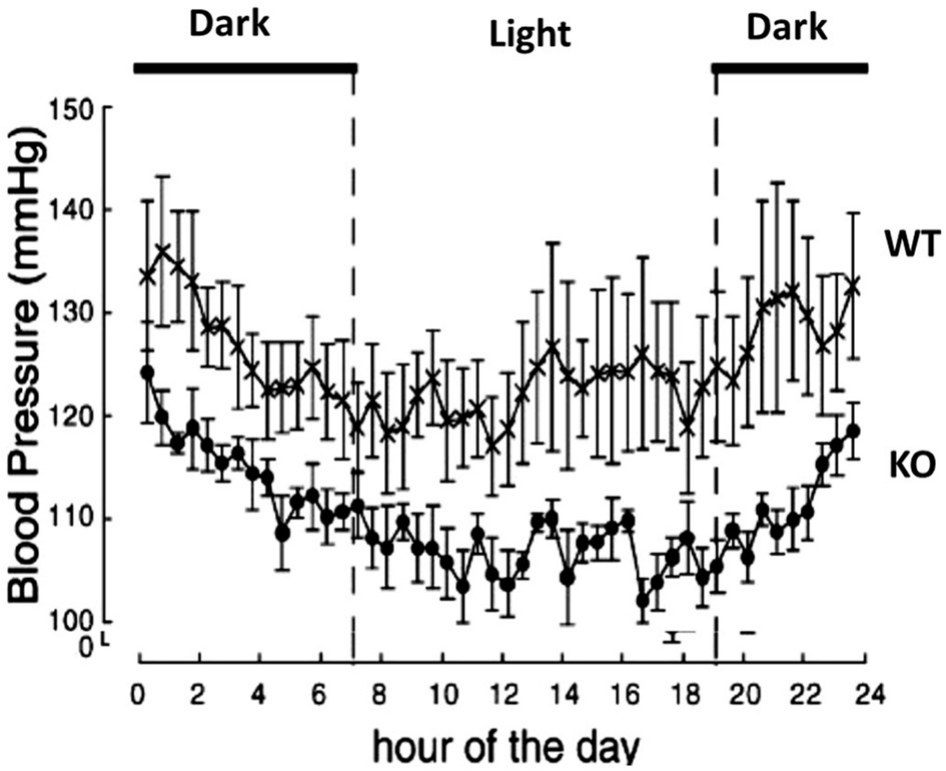
**Prepro-orexin knockout mice have lower blood pressures throughout the circadian cycle**. The knockout mice have lower blood pressure in both the light and dark periods of the diurnal cycle relative to the wild type control mice. Filled circles: KO mice; crosses: WT mice. (Figure used with permission from Kayaba et al., 2003).

**Figure 4:**
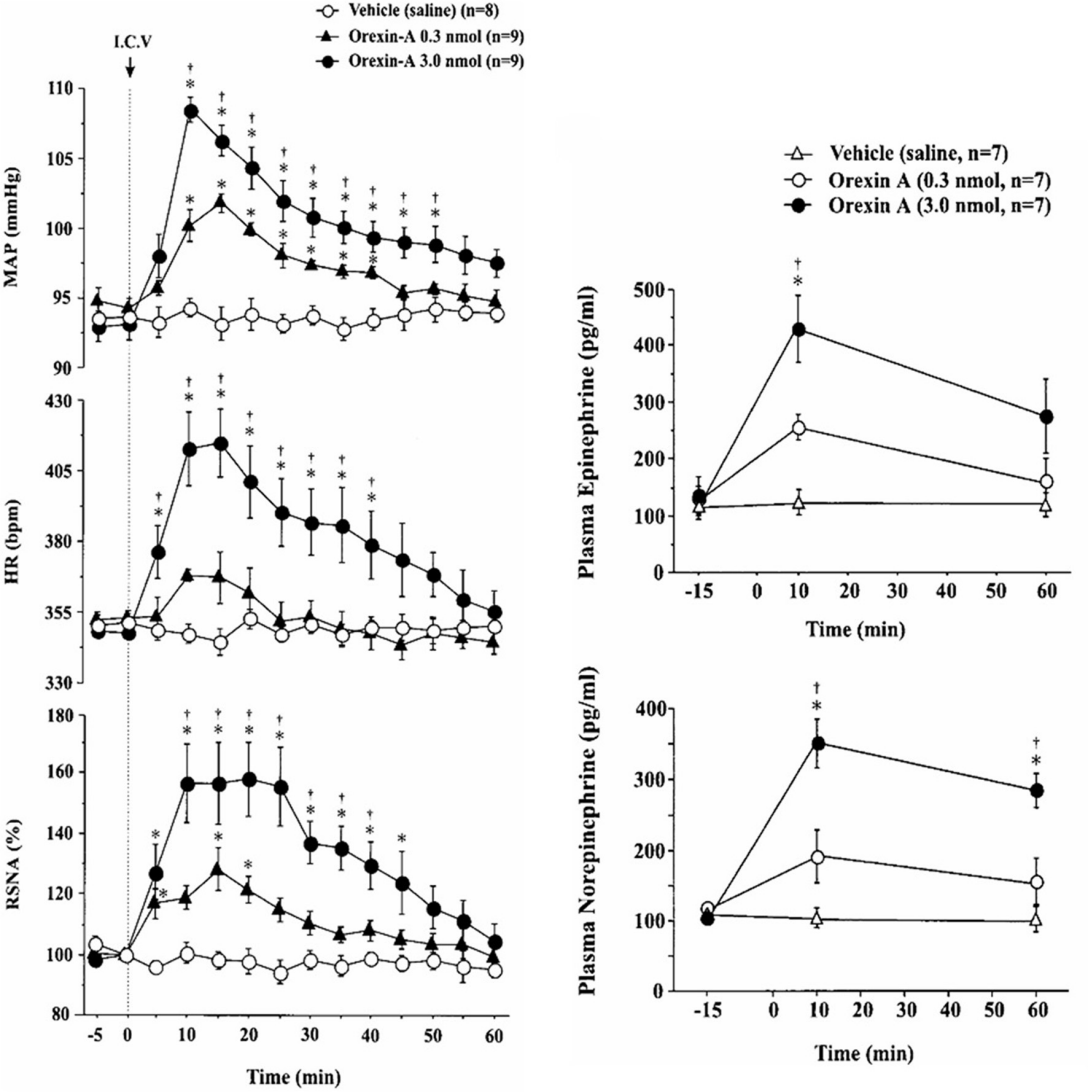
**Central application of OX-A increases mean arterial blood pressure (MAP), heart rate (HR) and renal sympathetic nerve activity (RSNA) in conscious rats**. Intracerebroventricular injection of OX-A increases MAP, RSNA, HR, and catecholamine release in conscious rats in a dose dependent manner. Orexin-A (0.3, 3.0 nmol) provoked an increase in MAP (94.3 ± 0.7 to 101.9 ± 0.7 mmHg and 93.1 ± 1.1 to 108.3 ± 0.8 mmHg, respectively) and RSNA (28.0 ± 7.0 and 57.9 ± 12.3%), respectively. (Figure used with permission from Shirasaka et al., 1999). **Left panel:** ^*^P < 0.05 vs. vehicle; ^†^*P* < 0.05 vs. orexin-A (0.3 nmol). **Right panel:** ^*^P < 0.05 vs. pre-injection values; ^†^*P* < 0.05 vs. orexin-A (0.3 nmol).

Functional studies *in vivo* have demonstrated that orexin participates in blood pressure regulation. Transgenic orexin deficient animals, both prepro-orexin knockout (pp-OX KO) mice (Figure 3) and rats with orexin neurons-genetically ablated (orexin/ataxin-3), have lower resting blood pressure (Kayaba et al., 2003; Schwimmer et al., 2010) relative to their wild type controls. In both anesthetized and unanesthetized normotensive animals, central administration of OX-A or OX-B activates sympathetic activity and increases both arterial blood pressure (ABP) and HR (Samson et al., 1999; Shirasaka et al., 1999, 2002; Chen et al., 2000; Antunes et al., 2001; Matsumura et al., 2001; Machado et al., 2002; Shahid et al., 2011, 2012). In conscious rats and rabbits (Shirasaka et al., 1999; Matsumura et al., 2001; Samson et al., 2007) central administration (icv) of orexin increases ABP (Samson et al., 1999; Shirasaka et al., 1999; Matsumura et al., 2001), and SNA (Shirasaka et al., 1999; Matsumura et al., 2001) in a dose dependent manner, and the increased ABP, HR and SNA induced by orexin is accompanied by an increase in plasma catecholamines (Figure 4) (Shirasaka et al., 1999; Matsumura et al., 2001). Intravenous injection of a ganglionic-blocking agent, pentolinium, can abolish OX-A induced increases in ABP and plasma epinephrine concentrations, which suggests that the pressor response induced by the icv injection of orexin-A can be attributed primarily to enhanced sympathetic outflow (Matsumura et al., 2001). Focal injection of OX-A into the RVLM can also induce a significant increase in ABP and HR in both anesthetized (Chen et al., 2000; Shahid et al., 2012) and conscious (Machado et al., 2002) rats. In anesthetized rats, the increased ABP is accompanied by increased splanchnic SNA(sSNA) and ventilation (phrenic nerve activity) that can be attenuated by blocking OX_1_R (Shahid et al., 2012). The peak effects following OX-A injection into the RVLM were observed at a dose of 50 pmol with ~42 mmHg increase in ABP and 45% increase in SNA (Shahid et al., 2012).

Other studies also directly and indirectly support orexin's role in the regulation of blood pressure and SNA, e.g., intrathecal injection of OX-A elicits a dose-dependent increase in ABP, HR (Antunes et al., 2001; Shahid et al., 2011), and sSNA (Shahid et al., 2011), and the effects can be partially attenuated by either beta-adrenergic or alpha-adrenergic receptor antagonists (Antunes et al., 2001) or OX_1_R antagonist (Shahid et al., 2011) in anesthetized rats. Microinjection of OX-A into the medullary raphe significantly increases ABP, HR, and body temperature in unanesthetized rats, (Luong and Carrive, 2012). It is well known that orexins are excitatory neuropeptides that also promote locomotion such as chewing and grooming (Shirasaka et al., 1999; Takakusaki et al., 2005; Thorpe and Kotz, 2005). To exclude the possible effects of increased locomotion on the changes in ABP, HR and SNA induced by the exogenous orexin in conscious rats, Shirasaka et al. injected OX-A icv in both anesthetized and conscious rats and tested them under the same experimental conditions. They found that OX-A induced a similar significant increase in ABP, HR and SNA in both anesthetized and conscious conditions in these rats (Shirasaka et al., 1999), which suggests that the sympathoexcitatory effects induced by exogenous orexin in the CSN are not due to the activation of locomotion.

Orexin is involved in the cardio-respiratory responses to acute stress, e.g., panic and fear (Kuwaki et al., 2008; Furlong et al., 2009; Iigaya et al., 2012; Johnson et al., 2012; Xiao et al., 2013). For example, in rats, silencing the hypothalamic pp-OX (*Hcrt gene*) with RNAi or antagonizing OX_1_Rs can block blood pressure and HR responses to acute panic stress (Johnson et al., 2010). And antagonizing both orexin receptors can: (1) significantly reduce the “bicuculline” induced stress responses, e.g., hypertension, tachycardia, and renal sympathoexcitation (Iigaya et al., 2012); (2) decrease the fear induced pressor, tachycardic, and locomotor responses (Furlong et al., 2009); and (3) decrease the hypercapnic-induced respiratory chemoreflex without affecting resting breathing (Li and Nattie, 2010).

In summary, studies in normal animals have shown that the central orexin system participates in regulation of blood pressure, HR, and SNA. Activation of OXRs in the CNS, or only the area of RVLM, by exogenous orexin causes sympathetically mediated hypertension and tachycardia, which can be attenuated by OXR antagonists. Orexin is necessary for blood pressure, HR and SNA responses to certain stresses, e.g., panic and fear.

### Orexin and hypertension

**Figure 5:**
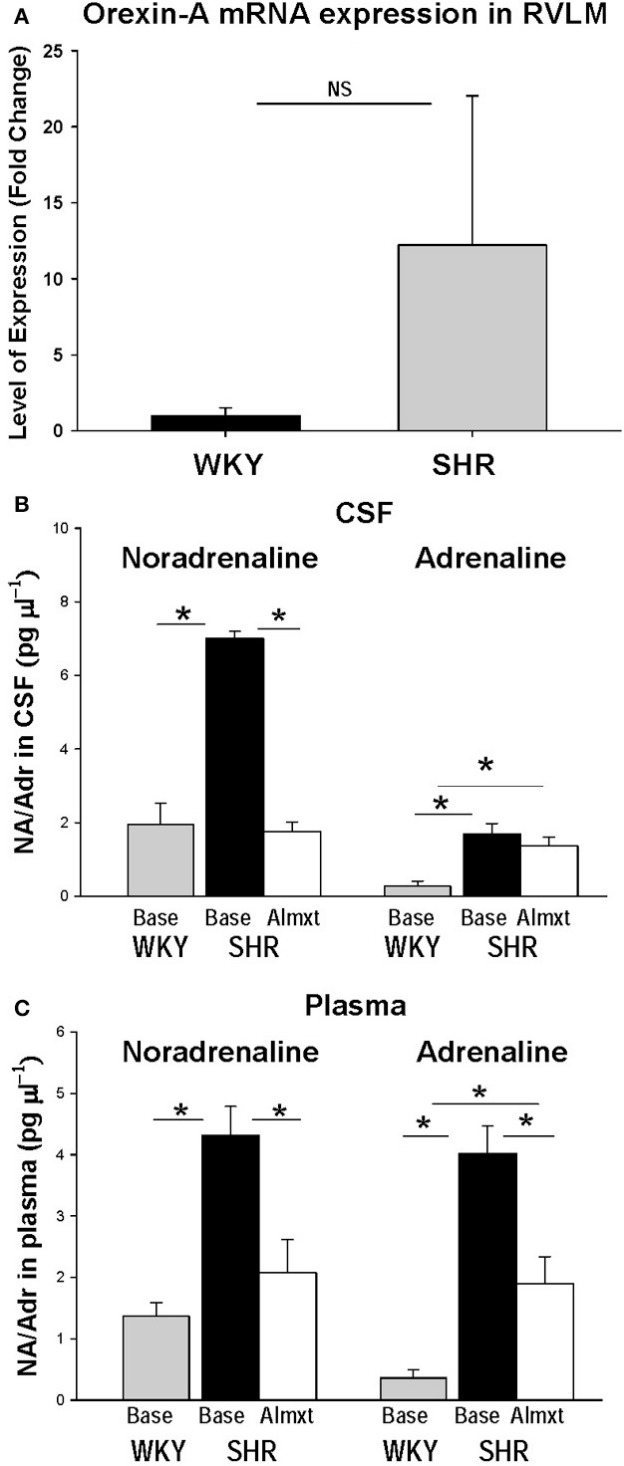
**In the RVLM of SHR, OX-A mRNA expression is increased as is noradrenaline in CSF and plasma**. There is clear trend toward an increase OX-A mRNA expression in RVLM in SHR relative to normotensive WKY rats **(A)**. Antagonism OXRs with Almxt significantly decreased the elevated levels of noradrenaline (NA) in CSF **(B)**, and both NA and adrenaline (Adr) in plasma in SHRs **(B,C)**. (Figure adapted with permission from Li et al., 2013a). ^*^Significantly difference (*P* < 0.002, One-Way ANOVA with Student–Newman–Keuls tests).

**Figure 6:**
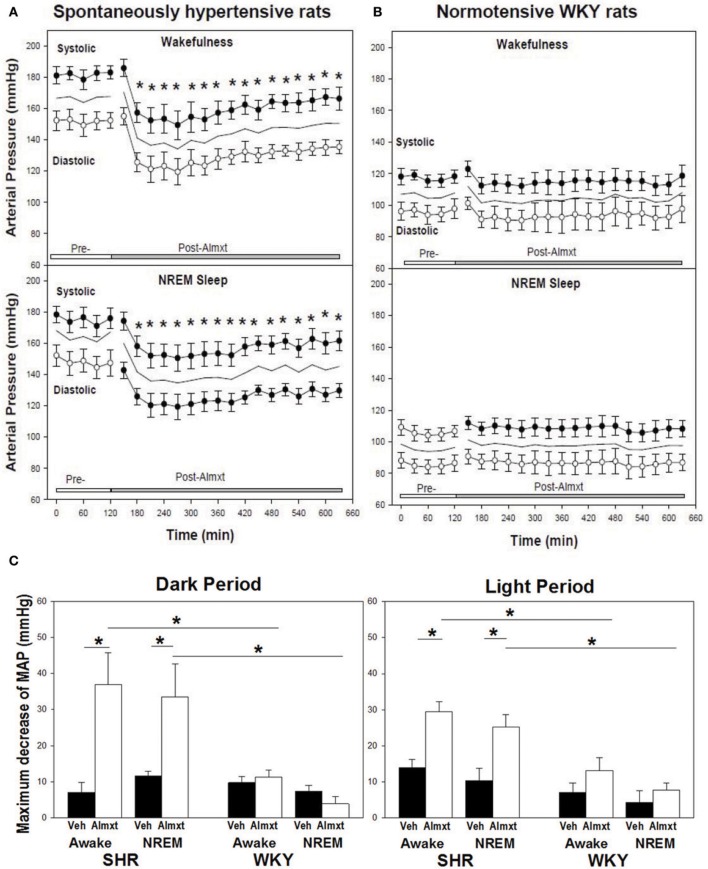
**In SHR, antagonism of OXRs with a systemic OXR antagonist, Almxt, decreases arterial pressure**. One does of orally administered amlxt significantly decreased arterial pressure in SHR for ~8 h **(A)**, and Almxt had no effect on arterial pressure in normotensive WKY rats **(B)**. The largest decrease of BP after Almxt is in wakefulness during the dark period (−37 mmHg) and smallest change is in NREM sleep during the light period (−25 mmHg) relative to the pretreatment baseline **(C)**. ^*^P < 0.02 (Figure adapted with permission from Li et al., 2013a).

**Figure 7:**
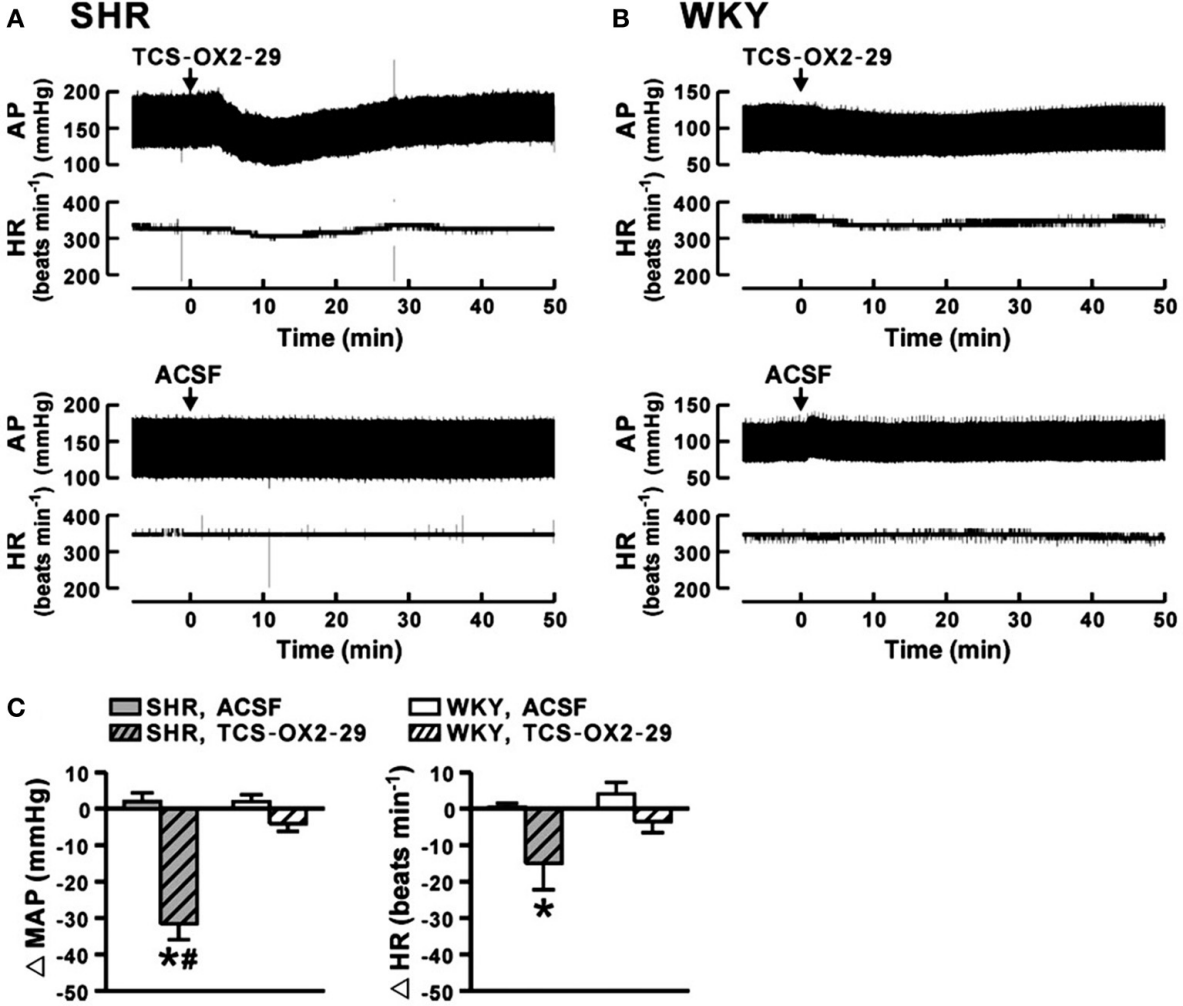
**In anesthetized SHR, antagonism of OX_2_R in RVLM decreases arterial pressure**. Microinjection of an OX_2_R antagonist (TCS-OX2-29) into the RVLM significantly decreased mean AP and HR in anesthetized SHR **(A,C)**, and the maximum change of mean AP and HR being 30 mmHg and 20 bpm respectively (C, gray hatched bars). It is important to note that blocking OX_2_R in RVLM in normotensive WKY rats had no effect on ABP and HR (B,C, white hatched bars). Control vehicle (ACSF) injection had no effect on AP and HR in SHR and WKY rats **(A,B,C)**. (Figure adapted with permission from Lee et al., 2013). ^*,#^Significant difference (*p* < 0.05).

The observations that: (a) orexin participates in the regulation of cardiovascular homeostasis, (b) exogenous orexins can increase SNA and blood pressure in normal animals, and (c) transgenic orexin deficient animals have lower resting blood pressure, have led two independent groups to test the hypothesis that orexin may participate in the development of neurogenic hypertension in spontaneously hypertensive rats (SHRs) (Lee et al., 2013; Li et al., 2013a). The SHR is one of the most studied animal models of neurogenic hypertension. As in human essential hypertension, the blood pressure of the SHR rises with age, starting at about 6 weeks, accompanied by an overactive sympathetic nervous system (Smith and Hutchins, 1979; Zicha and Kunes, 1999; Simms et al., 2009). We found (Li et al., 2013a,b) in unanesthetized freely moving adult SHRs that: (1) there is a strong trend toward a significant increase in orexin-A mRNA expression in the RVLM (Figure 5A), a projection site for orexinergic LHA neurons; (2) blocking both orexin receptors by oral administration of an antagonist, Almxt: (a) significantly lowers blood pressure in wakefulness and sleep during both the dark and light periods of the diurnal cycle, (b) the largest average decrease of ABP after blocking orexin receptors was in wakefulness during the dark period (−37 mmHg) and the smallest average change in NREM sleep during the light period (−25 mmHg) relative to the pretreatment baseline, (c) one dose of Almxt produced a remarkable and long-lasting (~8 h) reduction of ABP in SHRs (Figure 6); (3) the anti-hypertensive effect was accompanied by: (a) a significantly decreased SNA assessed by power spectral analysis of systolic ABP, and (b) decreased noradrenaline levels in cerebrospinal fluid and plasma (Figures 5B,C); (4) antagonizing orexin receptors had no effect on resting blood pressure in normotensive WKY rats (Figure 6B). Our findings are supported by a recent publication (Lee et al., 2013), which showed that blocking OX_2_R centrally by microinjection of an OX_2_R antagonist, TCS-OX2-29, into either the cerebral ventricle (icv) or the RVLM in anesthetized SHRs significantly decreased blood pressure (Figure 7) (Lee et al., 2013). The significant decrease in ABP following TCS-OX2-29 was observed at 3, 10, and 30 nmol doses, and the maximum reduction of ABP was ~21 or ~30 mmHg at 30 nmol with icv or RVLM injection respectively. It is important to note that in both studies antagonism of orexin receptor(s) with either Almxt or TCS-OX2-29 had no significant effect on ABP in conscious or anesthetized normotensive Wistar- Kyoto (WKY) control rats.

There are data on cardiovascular effects of the orexin system obtained from transgenic animals and hypertensive animal models that directly and indirectly support an orexin link to hypertension. Two recent genetic analysis studies showed that orexin related genes are altered in both SHRs and mice (Schlager BPH/2J) (Marques et al., 2011a,b; Yamamoto et al., 2013). Like SHRs, the Schlager high blood pressure mouse (BPH/2J) is a genetic model of neurogenic hypertension. Using affymetrix GeneChip mouse gene arrays Marques et al. (2011b) identified many genes that are differentially expressed in the hypothalamus of the hypertensive mouse relative to the normotensive BPN/3J control mice. Among these altered genes, the pp-OX (Hcrt) gene, which encodes orexin/hypocretin, is significantly increased or up-regulated in the hypothalamus in the hypertensive mice. BPH/2J hypertensive mice also exhibit a larger variation in ABP between the active and inactive periods of the day relative to the normotensive BPN/3J mice, and GeneChip mouse gene arrays showed that the pp-OX/Hcrt gene expression is higher during the active period when ABP is highest than in the inactive period when ABP is lowest in BPH/2J hypertensive mice, and is higher than that of in the normotensive control mice during the same period (Marques et al., 2011a). These genetic and functional studies in the neurogenic hypertensive animal models suggest that an up-regulated or overactive central orexin system may play an important role in developing and maintaining high blood pressure in neurogenic hypertension. It is also interesting to note that Yamamoto et al (Yamamoto et al., 2013) reported that the OX_1_R (Hcrtr1) gene is down-regulated in the adrenal gland in SHR and stroke-prone SHR (SHRSP) relative to normotensive WKY rats. This raises an interesting question of the role of peripheral orexin on cardiovascular function. As mentioned above, orexin neurons are exclusive located in the lateral hypothalamus and send projections to many brain locations. However orexins are found in many peripheral tissues, and the peripheral orexin distributions and functions have been discussed by recent reviews (Kukkonen et al., 2002; Spinazzi et al., 2006; Heinonen et al., 2008; Leonard and Kukkonen, 2013). Orexin receptors have been detected in various peripheral tissues, e.g., the gastrointestinal tract, pancreas, kidney, lung, adrenal gland, and adipose tissue (Kirchgessner and Liu, 1999; Lopez et al., 1999; Johren et al., 2001; Ouedraogo et al., 2003; Heinonen et al., 2008). In rat, both OX_1_R and OX_2_R mRNA can be detected in adrenal gland (Lopez et al., 1999; Johren et al., 2001), and the expression of OX_2_R mRNA in the adrenal is about eight times higher in male than that of female rats (Johren et al., 2001, 2004). The adrenal gland is importantly involved in stress-related cardiovascular function via the hypothalamic-pituitary-adrenal axis, and the significance of a down-regulated Hcrt1R gene in adrenal gland of SHRs needs to be further investigated.

Psychological stresses, e.g., anxiety, prolonged anger, mental stress, have long been considered as a contributing factor in developing hypertension (Zimmerman and Frohlich, 1990; Boone, 1991; Markovitz et al., 1993), and as discussed above orexin is involved in cardiovascular and respiratory responses to the acute stresses, e.g., panic, fear, drug or foot-shock induced, in animal models. Xiao et al. recently investigated possible role of orexin on developing stress related hypertension in adult Sprague-Dawley (SD) rats (Xiao et al., 2013). A stress induced-hypertensive rat (SIHR) was generated by intermittent electric foot-shocks (75–150 V, 0.5 ms duration) every 2–30 s and a buzzer noises (88–98 dB) for 2 h twice daily for 14 consecutive days. Adult normotensive SD rats develop hypertension after day 6 under these conditions. Using tail-cuff method the authors found the systolic blood pressure at 2 h after stress rose from 110 mmHg at baseline to 142 mmHg at day 14 in these SIHRs, and the number of OX-A immunoreactive (OXA-ir) neurons in the LHA and the protein level of OX_1_R in RVLM were significantly greater than that of the control rats. Microinjection of a selective OX_1_R antagonist (SB-408124), or a selective OX_2_R antagonist (TCS OX2 29) into RVLM can partially block the OX-A induced increased SBP and HR in SIHR (Xiao et al., 2013). It is interesting to note that, similar to the neurogenic hypertensive model SHRs, SIHRs also developed an overactive central orexin system. It would be interesting to see if blocking both OXRs by Almxt or a combination of OX_1_R and OX_2_R antagonists can decrease blood pressure between days 6 and 14 in these SIHRs and prevent the development of hypertension.

The potential peripheral effects of orexins on blood pressure and SNA have been investigated (Chen et al., 2000; Matsumura et al., 2001). Intravenous injection of OX-A or OX-B at a dose as high as 11 nmol/kg had no significant effect on ABP, HR or SNA in anesthetized rats and conscious rabbits, which suggests that the orexin effects on ABP, HR, and SNA are mediated primarily through the CNS (Chen et al., 2000; Matsumura et al., 2001). At the present time there is little evidence to support a role for peripheral orexins in the regulation of cardiovascular function (Heinonen et al., 2008; Kukkonen, 2013).

In summary: (1) Transgenic orexin deficient animals have a lower resting blood pressure. (2) Blocking OXRs significantly lowers blood pressure and SNA in adult SHRs. (3) PP-OX mRNA and gene expression are upregulated in the CNS in hypertensive rats and mice. Based on these findings we suggest that an overactive orexin system in the CNS may participate in the pathogenesis and maintenance of high blood pressure in certain forms of hypertension. Further, modulation of the orexin system could be a potential target in treating some forms of hypertension.

### Summary of the role of orexin in cardiovascular function

In normal animals, activation of OXRs by orexin in the CNS or in areas critically involved in SNA regulation, e.g., the RVLM and spinal cord, induces sympathetically mediated hypertension and tachycardia, which can be attenuated by OXR antagonists. Transgenic orexin deficient animals have a lower resting blood pressure. Spontaneously hypertensive rats and mice may have an overactive orexin system as (1) blocking OXRs produces significant anti-hypertensive effects in SHRs, (2) the expression of OX-A mRNA in increased in RVLM in SHRs and (3) the pp-OX/Hcrt gene is upregulated in hypothalamus in hypertensive BPH/2J mice. Orexin is important in the regulation of SNA and blood pressure and an overactive orexin system may be pathologically linked to the development of neurogenic hypertension.

## Orexin and respiration: the central chemoreflex and blood pressure regulation

### Orexin, respiration, and the hypercapnic chemoreflex

Anatomically the orexin system is well positioned to be involved in the regulation of respiration and central chemoreception. Orexin neurons innervate many brainstem respiratory nuclei including the RTN, medullary raphe, LC, NTS, and pre-Bötzinger complex (Peyron et al., 1998; Date et al., 1999; Kukkonen et al., 2002; Young et al., 2005; Puskas et al., 2010; Lazarenko et al., 2011; Tupone et al., 2011; Nixon et al., 2012). Retrograde tracing studies showed that LHA OX-A positive neurons project to the diaphragm (Young et al., 2005; Badami et al., 2010) and raphe pallidus (Tupone et al., 2011). In Phox2b-eGFP transgenic mice, Lazarenko et al. further showed that the orexin-containing axonal varicosities are closely positioned relative to RTN Phox2b expressing neurons (Lazarenko et al., 2011). The expression of OX_1_R and/or OX_2_R mRNA is also found abundantly in many respiratory nuclei in the brainstem with a pattern matching that of OX-containing axon terminals (Marcus et al., 2001; Kukkonen et al., 2002).

**Figure 8:**
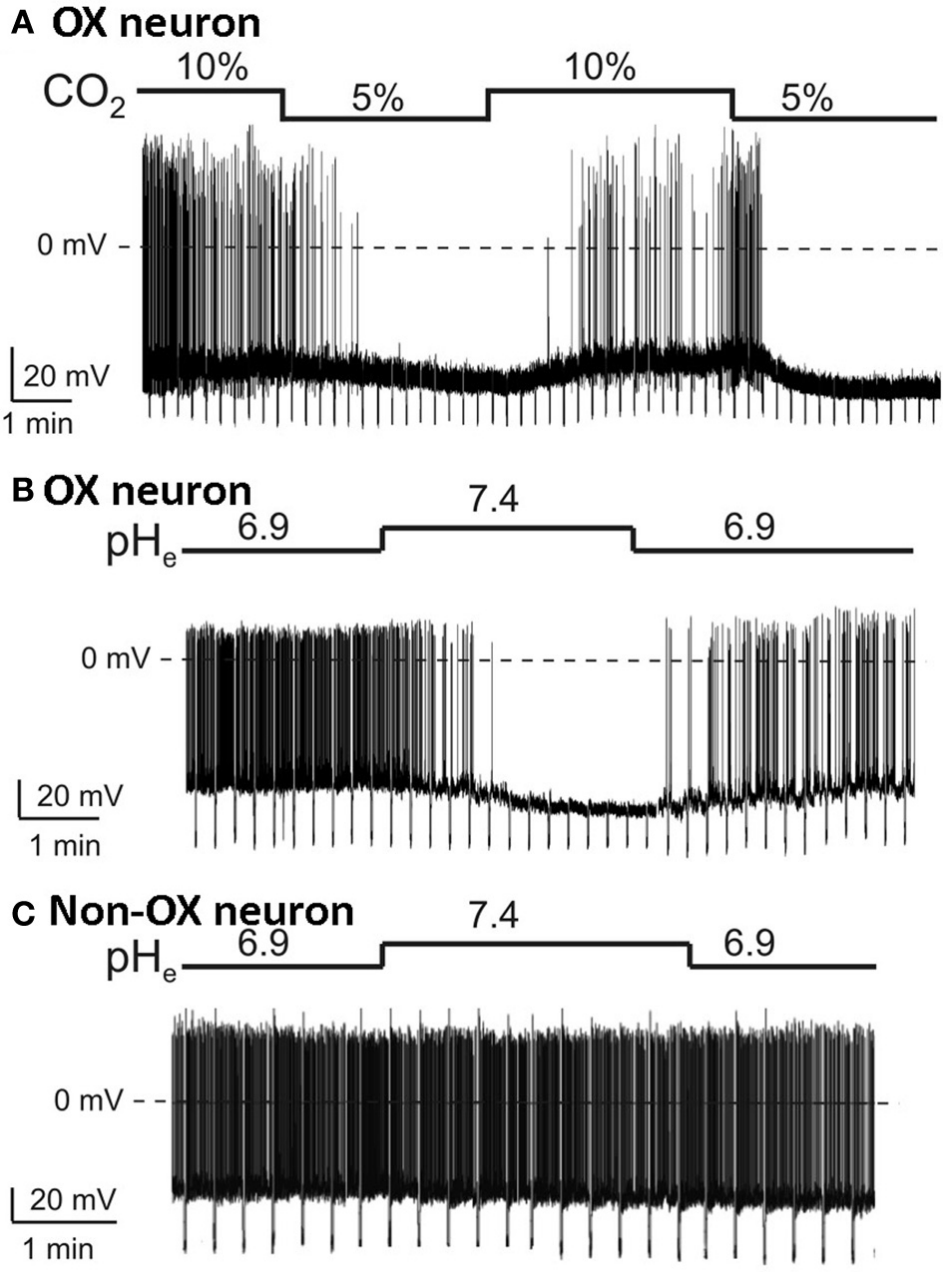
**Orexin neurons in LHA are intrinsically CO_2_ and pH sensitive**. *In vitro* current-clamp whole-cell recordings of GFP tagged orexin neurons of the Phox2b-eGFP mice show: **(A)** orexin (OX) neurons are intrinsically CO_2_ chemosensitive, Firing rate was 0.8 ± 0.3 Hz in 5% CO_2_ and increased to 3.7 ± 0.4 Hz in 10% CO_2_ (*n* = 5; *P* < 0.01); **(B)** orexin neurons are intrinsically pH chemosensitive. Firing rate was 4 ± 0.6 Hz at pH 6.9 and decreased to 0.5 ± 0.2 Hz at pH 7.4 (*n* = 15; *P* < 0.001); and **(C)** non-orexin neurons in the same region are not pH chemosensitive. (Figure used with permission from Williams et al., 2007).

At the cellular level, using *in vitro* patch-clamp recordings in visualized orexin neurons in brain slices, Williams et al., showed that the orexin neurons are intrinsically CO_2_ (Figure 8A) and pH (Figure 8B) sensitive (Williams et al., 2007). The orexin neurons in LHA increase their firing rate during acidification and decrease their firing rate during alkalization (Figure 8), while non-orexin neurons are not responsive to such changes (Figure 8C) (Williams et al., 2007). These effects are mediated, at least in part, by the TASK-like tandem-pore K^+^ channels (Williams et al., 2007), and possibly by other acid-sensing channels, e.g., ASICs (Song et al., 2012).

**Figure 9:**
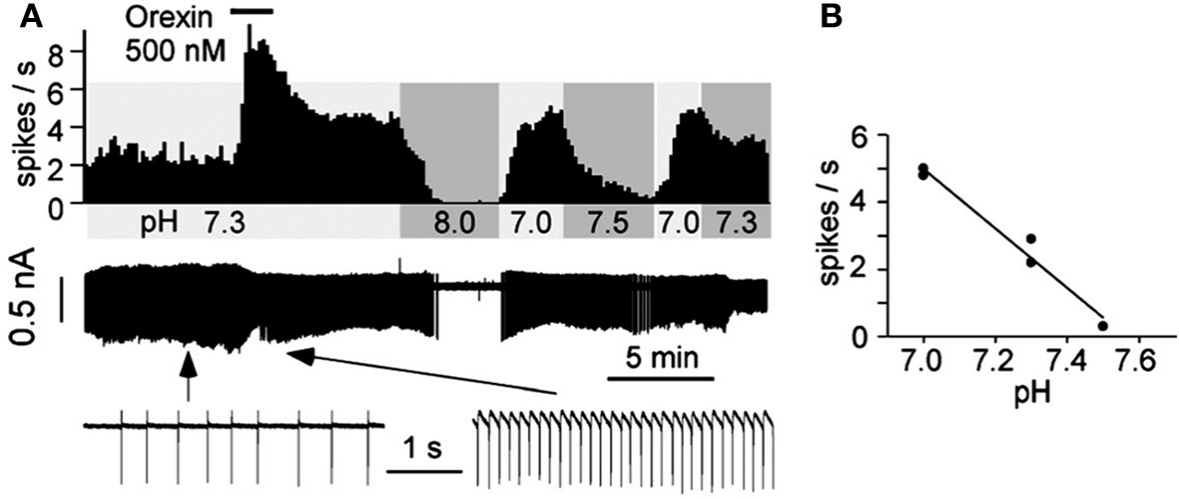
**Orexin A excites acid sensitive eGFP/Phox2b-expressing RTN neurons *in vitro***. Electrophysiological recordings in slices from neonatal (P6-P10) Phox2b-eGFP mice show that OX-A excited eGFP-expressing RTN neurons in a dose-related manner **(A)**. The neuron increased activity upon bath acidification, decreased firing with alkalization with an ~5 Hz dynamic range of response between pH 7.0 and 7.5 **(A,B)**. (Figure used with permission from Lazarenko et al., 2011).

**Figure 10:**
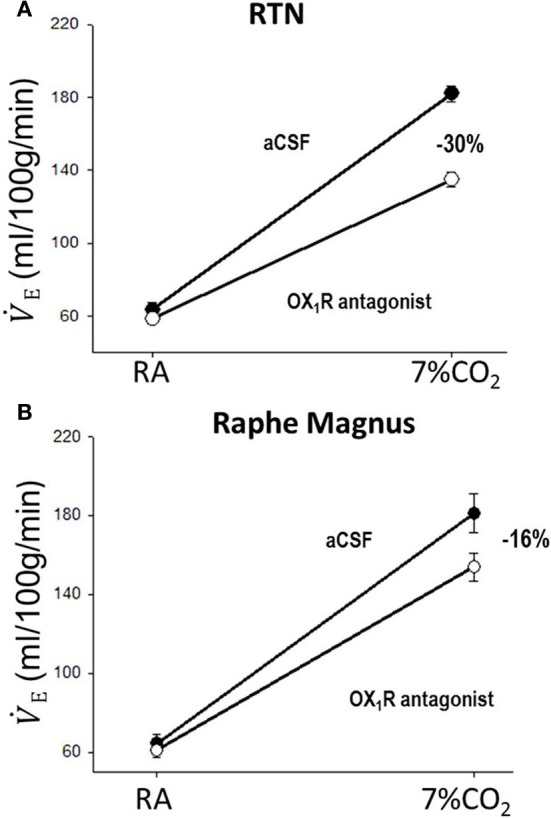
**Focal application of an OX_1_R antagonist in the RTN and the medullary raphe magnus decreases the CO_2_ chemoreflex**. Inhibition of OX_1_R in the region of RTN **(**unilateral, **A)** or raphe magnus **(B)** by an OX_1_R antagonist (SB334867) significantly decreased the CO_2_ chemoreflex by 30 or 16% respectively in wakefulness. (Figure adapted with permission from Dias et al., 2009 and Dias et al., 2010).

**Figure 11:**
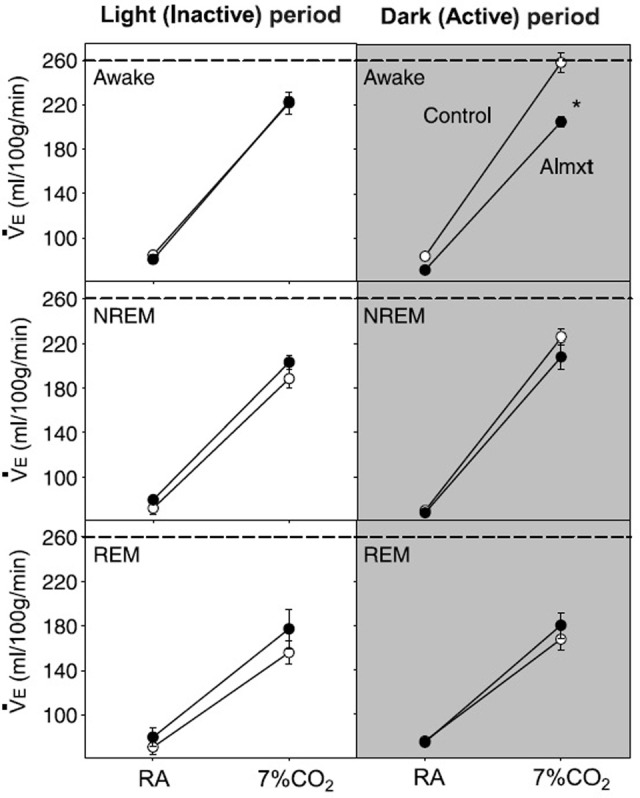
**Systemic antagonism of OXRs decreases the CO_2_ chemoreflex**. Antagonism of both OX_1_R and OX_2_R by orally administrating a dual OXR antagonist, Almxt, significantly decreased the hypercapnic chemoreflex only in wakefulness during the dark period of diurnal cycle (−26%). In the dark period, there was a significant difference between the ventilation breathing 7% CO_2_ in control compared to Almxt treatment in the awake state (^*^P < 0.05). (Figure used with permission from Li and Nattie, 2010).

In addition to their intrinsic CO_2_/pH sensitivity, orexin neurons also modulate the activity of brainstem chemosensitive neurons such as those in medullary raphe nuclei and RTN (Dias et al., 2010; Lazarenko et al., 2011; Tupone et al., 2011; Nattie and Li, 2012). Electrophysiological studies in slices from neonatal (P6-P10) Phox2b-eGFP transgenic mice showed that orexin A excites the acid sensitive eGFP/Phox2b-expressing RTN neurons in a dose dependent manner (ED50 ~250 nM, Figure 9), and these neurons increased activity upon bath acidification, decreased firing with alkalization, and exhibited an ~5 Hz dynamic range of response between pH 7.0 and 7.5 (Figures 9A,B) (Lazarenko et al., 2011). Functional studies have shown that the orexin system participates in the regulation of respiration and the CO_2_ central chemoreflex (Dutschmann et al., 2007; Nakamura et al., 2007; Williams et al., 2007; Dias et al., 2009, 2010; Li and Nattie, 2010; Lazarenko et al., 2011). Microinjection of orexin into the RVLM at the level of the pre-Bötzinger complex causes a significant increase in amplitude of integrated phrenic nerve activity (an index of tidal volume) in anesthetized and vagotomized rats (Young et al., 2005; Shahid et al., 2012). Injection of OX-B into the Kölliker–Fuse nucleus significantly increases breathing frequency in P21–42 day rats using the intra-arterially perfused working heart-brainstem preparation (Dutschmann et al., 2007). In the decerebrate cat, OX-A application into the hypoglossal motor nucleus increases genioglossus muscle activity (Peever et al., 2003). The pp-OX knockout mouse with a complete lack of orexin has normal resting breathing but a significantly attenuated respiratory chemoreflex in wakefulness compare to the wide type control mice and supplementation of orexins can partially restore the reflex (Deng et al., 2007; Nakamura et al., 2007). Unilateral administration of an OX_1_R antagonist (SB334867) in the RTN significantly reduced the respiratory response to hypercapnia (7%CO_2_) with a substantial effect in wakefulness (−30%; Figure 10A) and a much smaller effect in sleep (−9%) (Dias et al., 2009). In the medullary raphe, inhibition of the OX_1_R produced a significant reduction of the CO_2_ chemoreflex in wakefulness (−16%; Figure 10B) but not in sleep (Dias et al., 2010). Antagonism both OX_1_R and OX_2_R by orally administrating a dual OXR antagonist, Almxt, significantly decreased the hypercapnic chemoreflex only in wakefulness during the dark period of diurnal cycle (−31%); the CO_2_ chemoreflex was not significantly changed in sleep in the dark period and wakefulness and sleep in the light period of the diurnal cycles (Figure 11) (Li and Nattie, 2010). Antagonism of orexin receptors had no effect on resting breathing (Dias et al., 2009, 2010; Li and Nattie, 2010). These *in vitro* and *in vivo* experiments demonstrate that the orexin system is significantly involved in the control of breathing, particularly in the central CO_2_ chemoreflex.

In summary: (1) Orexin neurons are intrinsically chemosensitive and send projections to other central chemoreceptor sites, e.g., the RTN and medullary raphe. (2) Orexin may regulate breathing and central chemoreception directly and/or indirectly by recruiting other brainstem chemoreceptor sites, e.g., the RTN and medullary raphe. (3) Orexin modulation of the CO_2_ chemoreflex may be vigilance state dependent with the strongest effect being during wakefulness in the dark period. (4) Orexin may be a link between breathing and sleep-wake status.

### Respiration and central chemoreception in blood pressure regulation

#### Respiration, sympathetic activity, and blood pressure

Anatomically, many cardiovascular and respiratory related nuclei are closely intertwined within similar regions of the brain, or are even synaptically connected. Many of these nuclei are part of the respiratory-sympathetic network that is critical to the regulation of respiratory and sympathetic activity and blood pressure (Rosin et al., 2006; Zoccal et al., 2009a; Geerling et al., 2010; Guyenet et al., 2010). For example, in the brainstem, both the RVLM and the caudal ventrolateral medulla (CVLM), sites critically involved in the regulation of sympathetic tone, overlap with the ventral respiratory column including Bötzinger and pre-Bötzinger neurons. The neurons in the RTN, a putative central chemoreceptor site, innervate the entire ventrolateral medulla including the ventral respiratory column (VRC) and areas critically involved in the ABP and SNA regulation, e.g., the RVLM and CVLM (Rosin et al., 2006). The hypothalamic PVN, an important site for autonomic and endocrine homeostasis, is anatomically connected with many putative central chemoreceptors sites, e.g., the RTN, LC, NTS and medullary raphe (Geerling et al., 2010). The sympathetic preganglionic neurons (SPGNs) in the spinal cord receive prominent innervation from spinal interneurons, the RVLM, the midline medulla oblongata including medullary raphe serotonergic neurons, the pontine A5 noradrenergic cell group, the dorsolateral pons, the hypothalamic PVN, and the orexinergic neurons in the LHA (Jansen et al., 1995). The complex anatomical connections between the respiratory sites, central chemoreceptor sites and cardiovascular regulation sites suggest that the central respiratory chemoreflex is positioned to participate in the regulation or modulation of sympathetic activity and blood pressure.

#### Central chemoreception, SNA and blood pressure

**Figure 12:**
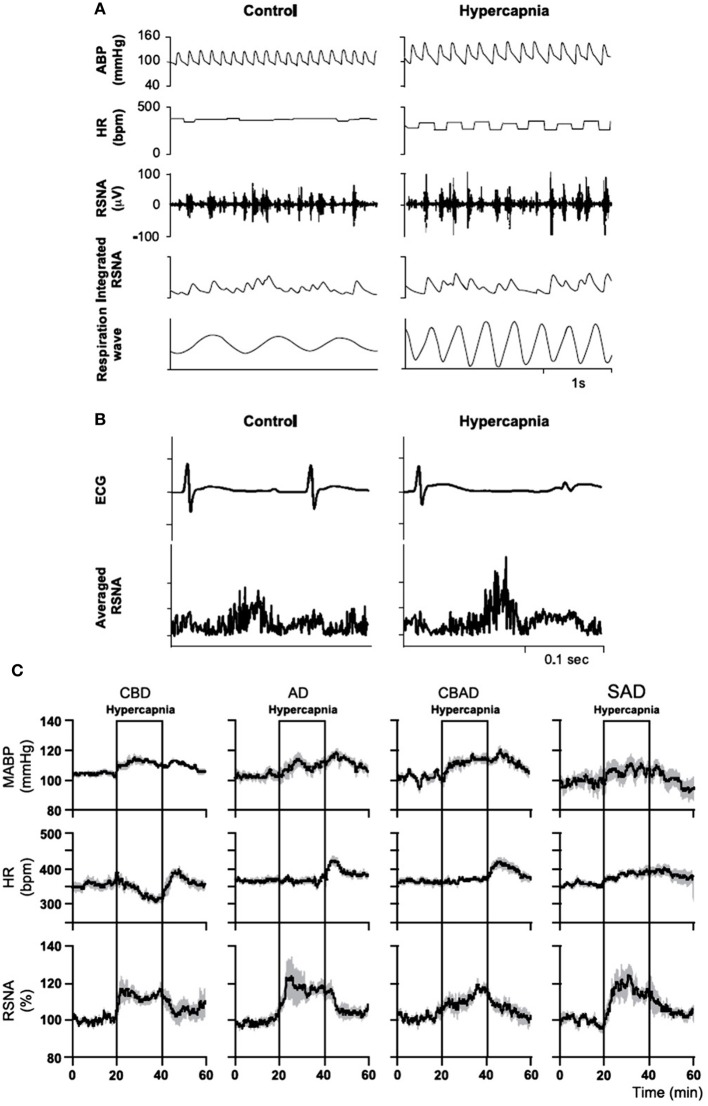
**In conscious rats, hypercapnia increases arterial blood pressure (ABP) and renal sympathetic nerve activity (RSNA)**. Hypercapnia increased mean ABP [from 105 to 117 mm Hg; **(A)**], RSNA [from 100 to 115%, **(A,B)**], and the respiratory rate [from 63 to 151 breaths/min; **(A)**], but decreased HR [from 374 to 302 beats/min; **(A,B)**]. Hypercapnia significantly increased mean ABP and RSNA in intact **(A)**, carotid body destroyed (CBD), aortic nerve denervated (AD), carotid body destroyed and aortic denervated (CBAD), and sinoaortic denervated (SAD) rats **(C)**. There were also no significant differences in the magnitudes of increase in ABP and RSNA during hypercapnia between the intact and the three chemo-denervated groups. (Figure adapted with permission from Oikawa et al., 2005).

Activation of central chemoreceptors by CO_2_/H^+^ increases ABP and SNA in both humans and experimental animals (Hanna et al., 1981; Lioy and Trzebski, 1984; Somers et al., 1989; Nattie et al., 1992, 1993; Oikawa et al., 2005; Guyenet et al., 2010). In humans, hyperoxic hypercapnia (7%CO_2_/93%O_2_) induced greater increases in ventilation ($\dot{V}$_*E*_), blood pressure and SNA than did hypoxia (10%O_2_/93%N_2_) (Somers et al., 1989). In conscious rats (Figure 12), Oikawa et al. (2005) showed that hypercapnia induced significant increases in mean ABP, RSNA, and the respiratory rate (Figure 12B) in intact animals and in animals with varied peripheral chemoreceptor input, e.g., carotid body destroyed (CBD), aortic nerve denervated (AD), carotid body destroyed plus aortic denervated (CBAD), and sinoaortic denervated (SAD) (Figure 12C). There were also no significant differences in the magnitudes of increase in ABP and RSNA during hypercapnia between the intact and the chemo-denervated groups (Figure 12). The fact that the increased ABP and rSNA response to hypercapnia was not affected by bilateral carotid chemo-denervation, aortic denervation, or sinoaortic-denervation suggests that the peripheral chemoreceptors do not play a major role in the cardiovascular response to hypercapnia in normal conscious rats (Oikawa et al., 2005). Blockade of glutamate receptors in the RTN or lesion of the rostral part of the RVLM decreased respiratory (phrenic nerve activity, PNA) and SNA responses to hypercapnic stimulation in decerebrate, paralyzed, vagotomized, and servo-ventilated cats (Nattie et al., 1992, 1993).

In summary: (1) Many putative central chemoreceptors sites, e.g., the RTN, NTS and medullary raphe, are involved in the regulation of SNA and blood pressure. (2) Activation of central chemoreceptors by hypercapnia significantly increases blood pressure, SNA and respiration with and without peripheral chemoreceptors. These studies suggest that the hypercapnic induced increase in ABP is due to sympatho-excitation via activation of the central chemoreceptors. The central chemoreceptors directly or indirectly regulate sympathetic vasomotor tone and blood pressure, and can activate the sympathetic outflow in a tonic manner independent of the effects on the central respiratory pattern generator (Moreira et al., 2006; Guyenet et al., 2010).

### Summary of orexin and respiration: the central chemoreflex and blood pressure regulation

Orexin neurons are intrinsically chemosensitive and their projections and receptors are found densely in all the major respiratory neuronal groups and central chemoreceptor sites. Activation of central chemoreceptors with and without activation of peripheral chemoreceptors leads to significant sympathoexcitation and hypertension in both human and experimental animals. Respiration and the central chemoreflex are involved in regulation of SNA and blood pressure.

## Orexin, chemoreflex, and hypertension

Overactive vasoconstrictor sympathetic tone, an enhanced peripheral chemoreflex, and an impaired baroreflex have been found in a significant portion of patients with primary hypertensive as well as in SHRs (Izdebska et al., 1982; Simms et al., 2009; Tan et al., 2010). In susceptible individuals, stress or altered physiology initiates increases in sympathetic activity to cardiovascular resistance vessels accompanied by increases in blood pressure. Over time, vascular smooth muscle in resistance vessels hypertrophies resulting in persistent hypertension. The SHR is one of the most used animal models of neurogenic hypertension and SHRs develop hypertension at about 6 weeks of life. Using the working heart–brainstem preparation, Simms et al., showed that the respiratory related sympathetic tone is significantly higher in SHRs relative to normotensive WKY rats starting from postnatal day 9–16, well before the onset of hypertension (Simms et al., 2009). They suggest that this augmented respiratory-sympathetic coupling in SHR and its effect on the vascular tone in early life is a causal factor in developing hypertension (Simms et al., 2009, 2010). However, the mechanisms leading to such changes remain unclear at present time.

### Peripheral chemoreflex and hypertension

**Figure 13:**
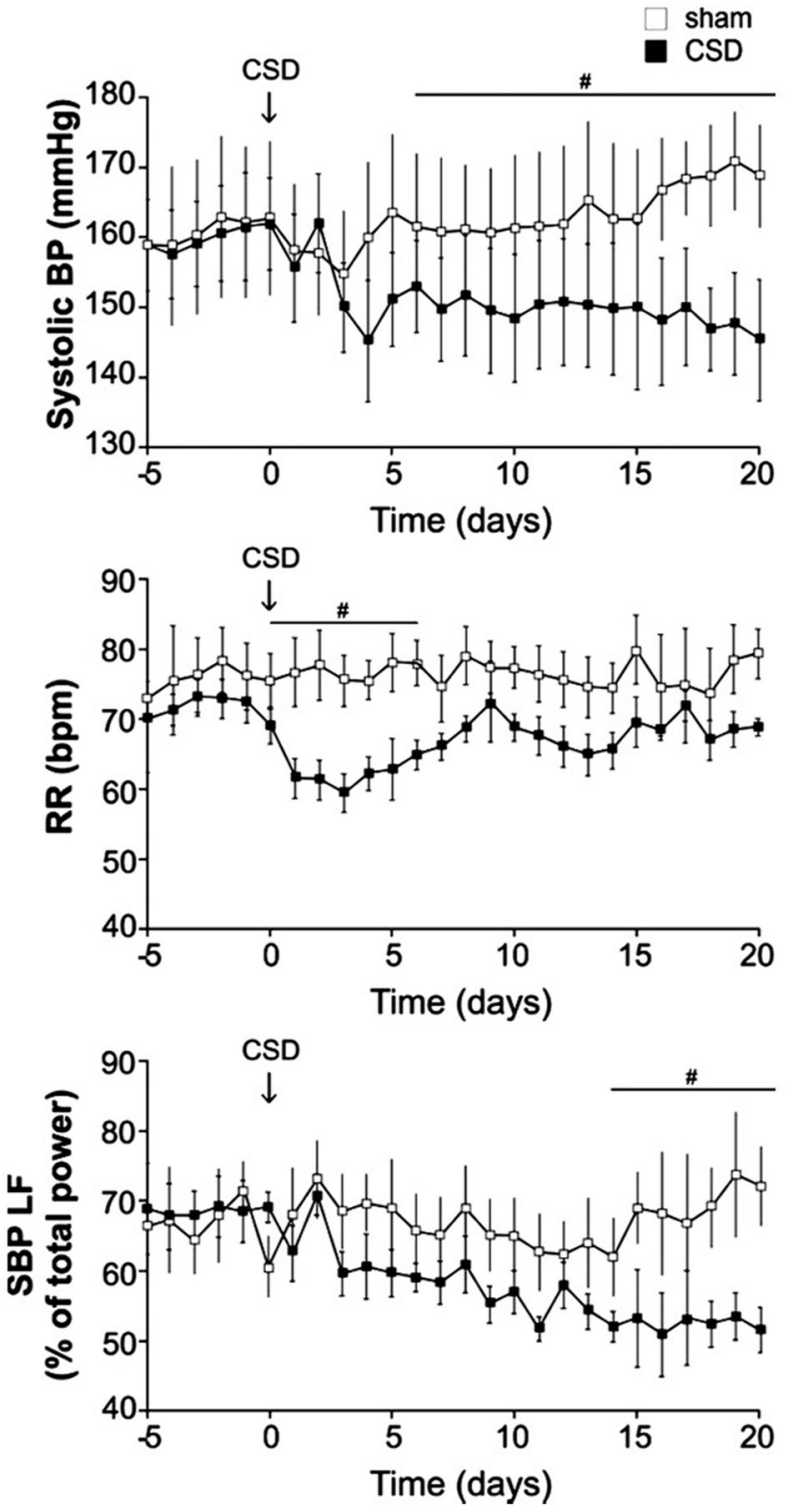
**In SHR, carotid body denervation decreases blood pressure, respiratory frequency and sympathetic nerve activity**. Bilateral denervation of carotid sinus nerves (CSD) significantly decrease resting blood pressure, respiratory frequency (RR; transiently) and a SNA index (SBP LF) in SHRs. Systolic pressure decreased over 5–10 days to reach a plateau of −17 ± 3 mmHg (*n* = 10, *P* < 0.05). Mean arterial and diastolic pressures fell by −15 ± 2 (*P* < 0.05) and −17 ± 2 (*P* < 0.05) mmHg, respectively. Lowered arterial pressure was maintained with no sign of recovery for at least 3 weeks. SBP LF: Systolic blood pressure, low frequency band. (Figure used with permission from Abdala et al., 2012). ^#^Significant difference (*P* < 0.05).

A link between neurogenic hypertension and an enhanced carotid body chemoreflex has been more extensively studied in patients and in animal models of sleep apnea (Fletcher et al., 1992; Lesske et al., 1997; Fletcher, 2001; Prabhakar et al., 2001, 2005, 2012; Schultz et al., 2007; Simms et al., 2010; Zoccal and Machado, 2011; Costa-Silva et al., 2012; Moraes et al., 2012a,b; Paton et al., 2013). The peripheral chemoreceptor reflex response has been shown to be significantly enhanced in patients with primary hypertension (Trzebski et al., 1982; Tafil-Klawe et al., 1985a,b; Somers et al., 1988a,b; Sinski et al., 2012) and in animal models of systemic hypertension, e.g., SHRs (Fukuda et al., 1987; Simms et al., 2009; Tan et al., 2010). Rats exposed to chronic intermittent hypoxia (CIH) develop hypertension and persistent sympathetic activation, and elimination of the carotid bodies prevents such CIH-induced hypertension (Fletcher et al., 1992; Lesske et al., 1997). Enhanced carotid body activity has been suggested to result in alterations in respiratory–sympathetic coupling (Simms et al., 2009; Zoccal et al., 2009b) and increased muscle vasoconstrictor activity, which may contribute to the development of hypertension (Trzebski et al., 1982; Somers et al., 1988a). The hyperactive carotid chemoreceptors are accompanied by overexpression of ASIC/TASK (acid-sensing ion channel/2-pore domain acid-sensing K^+^ channel) channels in young pre-hypertensive SHRs (Tan et al., 2010). Abdala et al., further showed that bilateral denervation of the carotid sinus nerves (CSD) in SHRs can significantly lower the resting blood pressure (~25 mmHg), respiratory frequency (transiently) and the low frequency component of the frequency analysis of systolic blood pressure, an index of sympathetic vasomotor tone (Figure 13), and they suggested that the inputs of carotid sinus nerve from the carotid body are partially responsible for increased SNA and blood pressure in SHR (Abdala et al., 2012).

In summary: an enhanced peripheral chemoreceptor reflex is found in both human hypertension and SHRs, and bilateral denervation of the carotid sinus nerves can partially lower blood pressure in SHRs. It is suggested that this enhanced carotid body activity may contribute to the alterations in respiratory–sympathetic coupling in SHRs.

### Central CO_2_ chemoreception, hypertension

As discussed above it is well established that activation of central chemoreceptors by CO_2_ increases breathing, SNA and blood pressure in humans and in anesthetized and conscious animals (Hanna et al., 1981; Lioy and Trzebski, 1984; Somers et al., 1989; Nattie et al., 1992, 1993; Oikawa et al., 2005; Guyenet et al., 2010). In asphyxia, the hypercapnic component is of greater importance than the hypoxic component in causing sympathetically induced increases in ABP and vascular resistance (Morgan et al., 1995; Cooper et al., 2004). However, the links between neurogenic hypertension and the hypercapnic central chemoreflex are not well understood. In human subjects, the hypoxic component of asphyxia reduces the baroreceptor–vascular resistance reflex sensitivity, while the hypercapnic component is responsible for increasing blood pressure (Cooper et al., 2004, 2005). Thus, the effects of both peripheral and central chemoreceptors may contribute to promoting hypertension in patients with obstructive sleep apnea who undergo repeated bouts of asphyxia nightly (Cooper et al., 2005). Combined hypoxia and hypercapnia evoke longer-lasting sympathetic activation in humans than does either hypoxia or hypercapnia alone (Morgan et al., 1995). In patients with heart failure, the central chemoreflex response to hypercapnia is markedly and selectively enhanced (Kara et al., 2003; Yamada et al., 2004), and the enhanced hypercapnic chemosensitivity is correlated significantly with plasma norepinephrine levels suggesting sympathoexcitation (Kara et al., 2003). Administration of 100% oxygen does not lower sympathetic activity in patients with heart failure, providing further evidence against any peripheral chemoreflex potentiation (Kara et al., 2003). In anesthetized SHRs with carotid sinus denervation, systemic hypercapnia increased and hypocapnia decreased the magnitude of both phrenic and sympathetic discharges, and the increased sympathetic discharge during hypercapnia was accompanied by a significant increase in ABP (Czyzyk-Krzeska and Trzebski, 1990). We have recently reported that SHRs have a significantly augmented central hypercapnic chemoreflex relative to the normotensive WKY control rats (Li and Nattie, Neuroscience, 2012). In SHRs, the ventilatory response to normoxic hypercapnia (7% CO_2_/21%O_2_), or hyperoxic hypercapnia (7%CO_2_/93%O_2_) when peripheral chemoreceptors are suppressed, is significantly higher in both wakefulness and sleep than that of the normotensive WKY control rats (Li and Nattie, Neuroscience, 2012). It is interesting to note that both peripheral and central chemoreflexes have powerful effects on sympathetic activity (see above), and in SHRs both the peripheral and central chemoreflex are overactive. Asphyxia, a recurring condition in sleep disorders, includes two important components, hypoxia and hypercapnia, which are primarily detected by peripheral and central chemoreceptors, respectively, in normal conditions. Sleep disorders are present in ~ 40% of obese individuals and both sleep disorders and obesity are closely associated with hypertension (Chau et al., 2012). Many studies have focused on the hypoxic effects of sleep disorders and hypertension with little attention to the role of the central CO_2_ chemoreflex in sleep disorders and their link to the development of hypertension. It is also interesting to note that while denervation of the peripheral chemoreceptor in SHRs significantly lower ABP (up to ~25 mmHg), ABP remains significantly above the normal even at 21 days post CSD (Abdala et al., 2012) suggesting other possible mechanisms are involved, e.g., an overactive central chemoreflex.

In summary: in SHRs, the respiratory chemoreflex to hypercapnia is exaggerated with and without peripheral carotid chemoreceptor inputs.

### Orexin, central chemoreceptors, and hypertension

That activation of orexin receptors evokes increases in breathing, SNA, and ABP, and that SHRs have augmented central and peripheral chemoreflexes suggest that orexin is a key factor that links the cardiovascular and respiratory systems, and that alterations of the orexin system may contribute to some cardio-respiratory diseases. The following observations support this proposed link: (1) The orexin knockout mouse has a decreased respiratory hypercapnic chemoreflex as well as a lower resting ABP (Kayaba et al., 2003; Nakamura et al., 2007). (2) Orexin injections into the CNS increase ABP, HR, SNA, and breathing in both conscious and anesthetized normotensive animals (Shirasaka et al., 1999; Machado et al., 2002; Huang et al., 2010; Shahid et al., 2011, 2012). (3) The SHRs have: (a) an augmented respiratory–sympathetic coupling in reduced preparations (Simms et al., 2009), (b) a hyperactive peripheral chemoreflex (Zoccal et al., 2008; Simms et al., 2009), and (c) a hyperactive central CO_2_ chemoreflex (Li and Nattie, Neuroscience, 2012). (4) Blockade of both OXRs can significantly lower ABP, HR, SNA and the hyperactive central CO_2_ chemoreflex in conscious SHRs (Li et al., 2013a). Based on these data, we pose the following questions: (1) What is the role of overactive central chemoreceptors in developing hypertension? (2) What is the role of orexin in this augmented CO_2_ chemoreflex and hypertension? (3) Can antagonism of OXRs be a target to treat such an augmented CO_2_ response and the associated hypertension in SHR? At present, we have found that antagonism of both OXRs using an orally administered dual orexin receptor antagonist, Almxt: (1) significantly reduced the exaggerated CO_2_ chemoreflex in SHR to the same level as measured in the control normotensive WKY rats (Li and Nattie, Neuroscience, 2012), and (2) significantly decreased ABP in both resting and hypercapnic condition (Li et al., 2013a) (Li and Nattie, Neuroscience, 2012). We hypothesize that the source of the enhanced SNA and respiratory-sympathetic coupling may involve neurons in the central chemoreceptor sites, and the overactive central orexin system may play an important role in such an alteration. There is no evidence that orexin projections and orexin receptors are located in the peripheral chemoreceptors, e.g., the carotid body, at the present time and the role of peripheral orexins in regulation of cardio-respiratory functions remain unclear (Johren et al., 2001; Nakabayashi et al., 2003; Heinonen et al., 2008).

In summary: transgenic orexin deficient mice and rats have lower resting blood pressure, and a significantly decreased hypercapnic response (pp-OX KO) while SHRs have severe hypertension, a hyperactive central hypercapnic reflex, and possibly an overactive orexin system. Blocking both orexin receptors in SHR can normalize the exaggerated hypercapnic chemoreflex and significantly lower blood pressure, which suggests the overactive central chemoreceptors may be an important link in neurogenic hypertension with orexin as mediator in SHRs.

### Summary of orexin, chemoreflex, and hypertension

Orexin knockout mice have both a severely attenuated hypercapnic chemoreflex and lower resting blood pressure, while SHRs have severely increased blood pressure and enhanced peripheral and central chemoreflexes with a possibly upregulated central orexin system. Antagonism of both orexin receptors can significantly lower SNA and blood pressure and normalize that central hypercapnic chemoreflex in SHRs. We suggest that overactive central chemoreceptors may be an important link to the development and maintenance of high blood pressure in SHRs with orexin as a key mediator.

## Conclusion

Activation of central CO_2_ chemoreceptors is associated with sympatho-excitation, hypertension and tachycardia. In SHRs, the overactive central and peripheral chemoreflex may play an important role in the development of neurogenic hypertension. Orexin links the respiratory and sympathetic nervous systems and an overactive orexin system may be the cause of hyperactive central CO_2_ chemoreflex in SHRs and thus the associated hypertension. Antagonism of OXRs can normalize the overactive CO_2_ central chemoreflex and significantly lower ABP and SNA in SHRs. Based on the data obtained from SHRs we hypothesize that modulation of the orexin system could be a potential target in treating some forms of hypertension.

### Conflict of interest statement

The authors declare that the research was conducted in the absence of any commercial or financial relationships that could be construed as a potential conflict of interest.
